# *Wushenziye* Formula Improves Skeletal Muscle Insulin Resistance in Type 2 Diabetes Mellitus via PTP1B-IRS1-Akt-GLUT4 Signaling Pathway

**DOI:** 10.1155/2017/4393529

**Published:** 2017-12-31

**Authors:** Chunyu Tian, Hong Chang, Xiaojin La, Ji-an Li

**Affiliations:** ^1^North China University of Science and Technology, Tangshan 063210, China; ^2^Pharmacology Analysis Key Laboratory for Prevention and Treatment of Diabetes of Traditional Chinese Medicine in Hebei Province, Tangshan 063210, China

## Abstract

*Background. Wushenziye* formula (WSZYF) is an effective traditional Chinese medicine in the treatment of type 2 diabetes mellitus (T2DM).* Aim.* This study aimed to identify the effects and underlying mechanisms of WSZYF on improving skeletal muscle insulin resistance in T2DM.* Methods.* An animal model of T2DM was induced by Goto-Kakizaki diabetes prone rats fed with high fat and sugar for 4 weeks. Insulin resistance model was induced in skeletal muscle cell.* Results. In vivo*, WSZYF improved general conditions and decreased significantly fasting blood glucose, glycosylated serum protein, glycosylated hemoglobin, insulin concentration, and insulin resistance index of T2DM rats.* In vitro*, WSZYF enhanced glucose consumption in insulin resistance model of skeletal muscle cell. Furthermore, WSZYF affected the expressions of molecules in regulating T2DM, including increasing the expressions of p-IRS1, p-Akt, and GLUT4, reducing PTP1B expression.* Conclusion*. These findings displayed the potential of WSZYF as a new drug candidate in the treatment of T2DM and the antidiabetic mechanism of WSZYF is probably mediated through modulating the PTP1B-IRS1-Akt-GLUT4 signaling pathway.

## 1. Introduction

Diabetes mellitus characterized by deregulation of glucose and lipid metabolism seriously affects human health. It affected an estimated 366 million people in 2011 and the number is projected to be 600 million by the year 2035 [1]. Type 2 diabetes mellitus (T2DM), one of the most common types of diabetes with the characteristics of insensitivity to insulin, has attracted great attention [2]. Insulin resistance (IR), an impaired biological response to insulin, is the pathological basis of T2DM. It refers to the decreased sensitivity of tissues to insulin, resulting in the reduction in glucose uptake and utilization [3]. In the body, skeletal muscle is the main target organ that consumes glucose and completes approximately 80% of the postprandial glucose intake and consumption caused by insulin stimulation [4, 5]. However, under the condition of IR, insulin-stimulated glucose disposal in skeletal muscle is severely damaged and could not respond to insulin properly. This leads to a defect in the insulin signaling pathway in muscle and elevating blood glucose level, which is a key feature of IR in T2DM. Therefore, skeletal muscle was selected as a therapeutic target of WSZYF in the battle against T2DM.

The main proteins in the phosphatidylinositol 3-kinase (PI3K) signaling pathway, including PI3K, Akt, and glucose transporter type 4 (GLUT4), play important roles in insulin signaling transduction. The abnormality of this pathway is the major cause of T2DM [6]. The signaling cascade is triggered when insulin connects with the membrane receptor of target cells. Insulin receptor substrate-1 (IRS-1) phosphorylated by the activated insulin receptor leads to PI3K and Akt activation [7]. GLUT4, downstream of PI3K, is the pivotal protein in controlling glucose uptake and glycogen metabolism [8]. In T2DM, not only is the sensitivity of skeletal muscle to insulin abated, but also the expression of GLUT4 is decreased, which decreases the glucose uptake and utilization and raises blood glucose level [9, 10]. Moreover, the lack activity of IRS-1 leads to the reduced phosphorylation of PI3K and thereby decreased GLUT4 expression in skeletal muscle cells, resulting in IR [11]. Thus, the modulation of IRS1-Akt-GLUT4 signaling pathway is quite associated with the treatment of IR.

Protein tyrosine phosphatase-1B (PTP1B), which leads to the dephosphorylation of insulin receptor, has the negative regulation in insulin signal transduction [12]. Studies [12–14] have demonstrated that inhibition of PTP1B could result in the phosphorylation of insulin receptor and insulin receptor substrates (IRS), thus improving insulin sensitivity and decreasing blood glucose level. Therefore, targeting on PTP1B may be a novel approach for the treatment of T2DM.

Traditional Chinese medicine has made significant contributions to the prevention and treatment of T2DM. WSZYF is an effective compound in the treatment of T2DM in clinic. It consists of four Chinese medicines, including Radix Polygoni Multiflori Preparata, Mori fructus, Mori folium, and Cassiae semen. It was testified that components in WSZYF, like resveratrol and 2-styrene glucoside [15, 16], had the effects on improving insulin sensitivity. The antidiabetic effect and underling mechanism of WSZYF have not been clearly explained. In this study, a T2DM rat model and an IR model of skeletal muscle cell were established to investigate whether WSZYF could improve glucose metabolism and IR or not and how to regulate PTP1B-IRS1-Akt-GLUT4 signaling pathway by WSZYF.

## 2. Materials and Methods

### 2.1. Materials

Insulin assay kit was obtained from Millipore (USA). Dulbecco's modified Eagle's medium (DMEM) and fetal bovine serum (FBS) were from Gibco (USA). Metformin was purchased from Squibb Pharmaceutical (Shanghai, China). Blood sugar meter and blood sugar test paper were purchased from Sannuo Biosensors (China). Assay kits of glycosylated serum protein and glycosylated hemoglobin were from Nanjing Jiancheng Bioengineering Institute (Nanjing, China). The primary antibodies against PTP1B, p-Akt (phospho S473), GLUT4, and GAPDH were from Bioworld Technology (USA) and antibody against p-IRS1 (phospho Y612) was from Abcam (USA). The secondary antibodies were purchased from Beyotime Biotechnology (China). The herbs in WSZYF were purchased from Tongrentang Pharmacy (Tangshan, China).

### 2.2. Animals and Experimental Design

All the procedures were conformed to the Guide for the Care and Use of Laboratory Animals published by the National Institutes of Health (NIH Publications number 85-23, revised 1996). Goto-Kakizaki (GK) rats weighing 200 to 300 g were purchased from Shanghai Slack Animal Center (certificate number SCXK (Shanghai) 2012-0002). Rats were raised in specific pathogen-free (SPF) room in North China University of Science and Technology. After one-week acclimation, rats were fed with high glucose and fat for 4 weeks to induce T2DM model. Rats with fasting blood glucose *⩾* 11.1 mmol/L and blood glucose *⩾* 16.7 mmol/L were randomly divided into five groups: model group (*n* = 6, oral administration of equivalent volume of normal saline), metformin group (*n* = 6, oral administration of metformin, 85 mg/kg/day), WSZYF low-dose group (WSZYF (L),* n* = 6, oral administration of WSZYF, 300 mg/kg/day), WSZYF medium-dose group (WSZYF (M),* n* = 6, oral administration of WSZYF, 600 mg/kg/day), and WSZYF high-dose group (WSZYF (H),* n* = 6, oral administration of WSZYF, 1200 mg/kg/day). The Wistar rats orally given equivalent volume of normal saline served as the control group. All the rats were raised for 8 weeks. At the end of the study, general conditions were measured and indexes related to blood glucose and insulin were tested. Muscle tissues were stored at −80°C for further analysis.

### 2.3. Body Weight, Food Intake, Water Intake, and Urine Output

The body weight, food intake, water intake, and urine output of the rats were monitored at the time points of 0, 1, 4, and 8 weeks and the data of 8 weeks were selected in this study. For urine output, rats were put into the metabolic cage after fasting for 12 hours. Urine in 24 hours was collected. During this time, they were only provided with food but no water.

### 2.4. Blood Chemistry Assay

After eight weeks, blood samples were collected for blood chemistry measurements. Fasting blood glucose was measured by using blood sugar meter and blood sugar test paper. Glycosylated hemoglobin, glycosylated serum protein, and insulin were detected by kits analysis according to manufacturer's instruction. Insulin resistance was assessed by a homeostasis model assessment of IR index as previously described [17].

### 2.5. Glucose Concentration in the Cell Supernatant Analysis

Primary cell culture of skeletal muscle was performed as previous study [18]. Muscle tissue was dissected from the soleus and cut into small pieces at the size of 0.1 cm^3^ and washed 3 times with PBS and DMEM (mixed with 10% FBS and 2% penicillin/streptomycin). Then the small pieces of muscle tissue were seeded in culture flasks which were upside down in in a humidified incubator with 5% CO_2_ at 37°C. After 2 hours, the culture flasks were right side up and 3–5 ml culture medium was added. The culture medium was renewed every three days, while cultures were monitored by daily observation under an inverted microscope (Olympus, Japan). Then the cells were purified by differential adherence and the second generation was used in the experiment. When the skeletal muscle cells reached 70% confluence, they were induced to differentiation by DMEM supplemented with 2% FBS, 100 U/ml penicillin, and 100 *μ*g/ml streptomycin. The successful differentiation showed that most of the myocytes had differentiated into multinucleated myotubes and could be easily identified as muscle cells. Then cells were divided into several groups: control group, model group, WSZYF-L (25 *μ*g/L) group, WSZYF-M (100 *μ*g/L) group, and WSZYF-H (400 *μ*g/L) group. In the model group, cells were washed three times with PBS and insulin (5 × 10^−7^ mol/L) with DMEM 200 *μ*l was added for 12 hours. Then the supernatant was removed and DMEM was added for 24-hour incubation. Cells in the treatment groups were treated in a similar way, except that the culture media contained WSZYF in the later 24 hours. In the control group, fresh nutrient solution was changed at the time points of 12 and 24 hours.

### 2.6. Western Blotting Analysis on Proteins Related to Regulating Insulin Resistance

Proteins related to regulating insulin resistance were detected by* in vivo* study. The collected muscle tissues (120 mg) were prepared with RIPA buffer (PPLYGEN, China) and proteins were extracted according to the manufacturer's instruction. Protein contents were measured with BCA protein assay kit (PPLYGEN, China). After addition of loading buffer boiled for 5 minutes, tissue samples were separated by 10% SDS-PAGE and transferred to NC membranes (Millipore, Germany). After being blocked with 5% nonfat dry milk for 2 hours, the membranes were incubated with different primary antibodies overnight at 4°C. After washing with TBST three times, the membranes were incubated with HRP-conjugated secondary antibodies for 1 hour at room temperature. Washed three times with TBST, the proteins were detected with an enhanced chemiluminescence agent (GE, USA) and quantified by densitometry using an image analyzer (Bio-Rad, USA). Mouse anti-GAPDH monoclonal antibody served as an internal control.

### 2.7. Statistical Analysis

Data were expressed as the mean ± standard deviation (SD). Statistical analysis was undertaken by one-way analysis of variance (ANOVA) and Dunnett's test. Differences between groups were considered as statistically significant when* P* < 0.05.

## 3. Results

### 3.1. Effects of WSZYF on General Conditions in T2DM Rats

**Figure 1:**
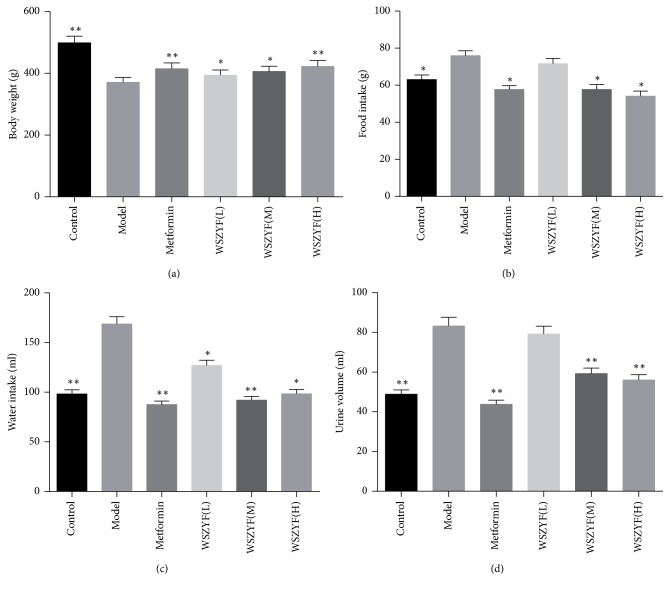
Effects of WSZYF on general conditions in T2DM rats. (a) Body weight, (b) food intake, (c) water intake, and (d) urine volume. ^*∗*^*P* < 0.05, ^*∗∗*^*P* < 0.01 compared with model group.

Food and water intake and urine output in the model group were significantly higher and body weight was lower than that of control group. Treatments of WSZYF (M, H) and metformin increased body weight and decreased food and water intake and urine output compared with the model group. These results indicated that WSZYF could ameliorate the general conditions of T2DM rats. Low dose of WSZYF had no significance in food intake or urine volume (Figure 1).

### 3.2. Effects of WSZYF on Glucose Metabolism Disorders

**Figure 2:**
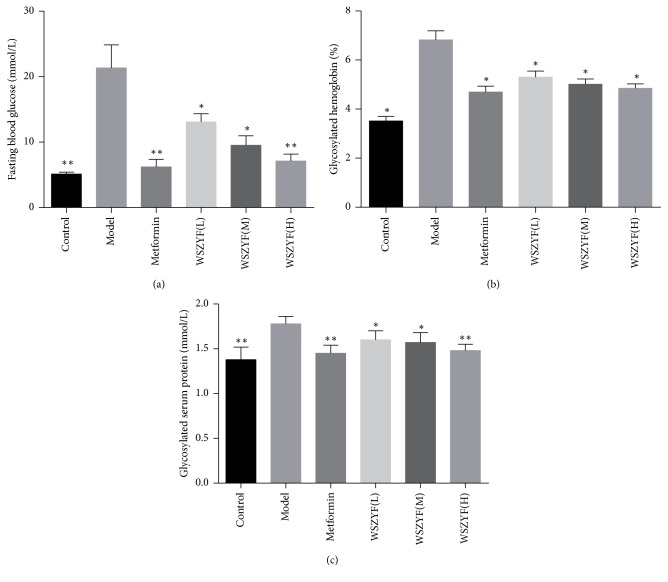
Effects of WSZYF on fasting blood glucose, glycosylated hemoglobin, and glycosylated serum protein. (a) Fasting blood glucose, (b) glycosylated hemoglobin, and (c) glycosylated serum protein. ^*∗*^*P* < 0.05, ^*∗∗*^*P* < 0.01 compared with model group.

As shown in Figure 2, fasting blood glucose, glycosylated hemoglobin, and glycosylated serum protein increased significantly in T2DM model group. These results suggested that glucose metabolism was disrupted. Treatments of WSZYF and metformin lowered all the three indexes mentioned above, indicating that they could restore glucose metabolism disorders.

### 3.3. Effects of WSZYF on Insulin Sensitivity

**Figure 3:**
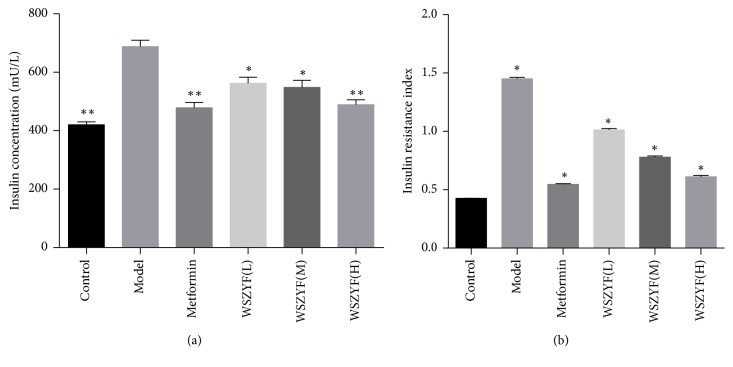
Effects of WSZYF on insulin and IR index. (a) Insulin concentration and (b) IR index. ^*∗*^*P* < 0.05 and ^*∗∗*^*P* < 0.01 compared with model group.

Insulin plays a critical role in the maintenance of blood glucose homeostasis. Further studies displayed the effects of WSZYF on insulin sensitivity. Insulin concentration and IR index increased in model group, which proved the IR occurrence. After treatments, these two indicators were decreased to be normal, demonstrating that WSZYF had the ability to enhance insulin sensitivity (Figure 3).

### 3.4. Effects of WSZYF on Glucose Consumption

**Figure 4:**
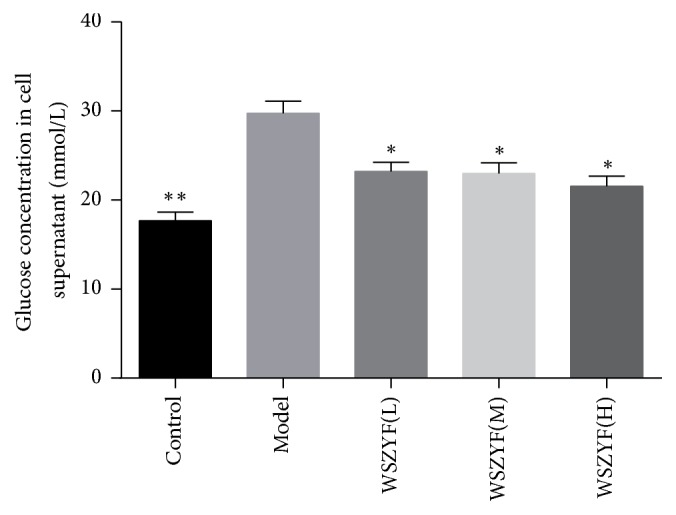
Effects of WSZYF on glucose consumption. ^*∗*^*P* < 0.05 and ^*∗∗*^*P* < 0.01 compared with model group.

In order to test the effect of WZSYF on glucose uptake, an IR model of skeletal muscle induced by insulin stimulation was established. In Figure 4, the glucose concentration in the cell supernatant of model group was higher than that of control group, indicating that glucose consumption was decreased in model group. Treatments of WZSYF decreased glucose concentration in the cell supernatant, which proved WSZYF could enhance glucose uptake of skeletal muscle cells.

### 3.5. Effects of WSZYF on Expressions of Insulin Signaling Transduction-Related Proteins

**Figure 5:**
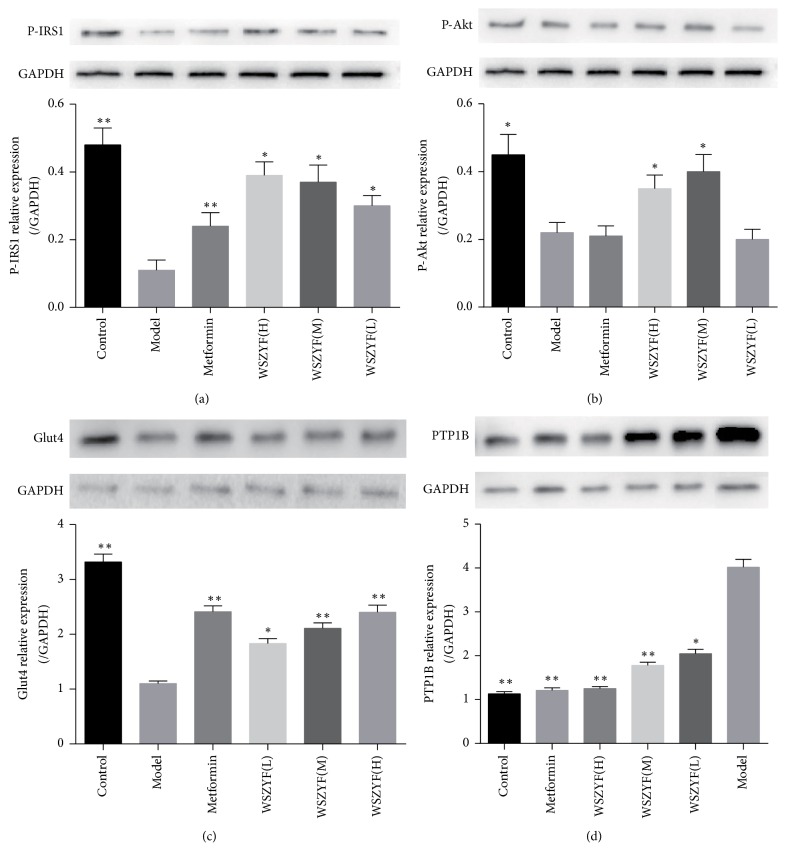
Effects of WSZYF on proteins related to insulin signal transduction. (a) P-IRS1 relative expression, (b) P-Akt relative expression, (c) GLUT4 relative expression, and (d) PTP1B relative expression. ^*∗*^*P* < 0.05 and ^*∗∗*^*P* < 0.01 compared with model group.

We further investigated the mechanism of WSZYF in the regulation of insulin signal transduction. As shown in Figure 5, the expressions of P-IRS1, P-Akt, and GLUT4 decreased, whereas PTP1B expression increased in model group compared with control group, indicating that insulin signal transduction was disrupted in T2DM rats. Expressions of these proteins were reverted to normal levels in treatment of WSZYF and metformin. The results showed that the antidiabetic effect of WSZYF may be mediated by modulating PTP1B-IRS1-Akt-GLUT4 anti-insulin resistant signaling pathway.

## 4. Discussion

Insulin resistance of skeletal muscle has been shown to play a vital role in the pathogenesis of T2DM. In this study, we demonstrated that WSZYF could exert antidiabetic effect by improving IR in skeletal muscle. Further* in vivo* study suggested that the anti-insulin resistant effect of WSZYF was potentially triggered by regulating PTP1B-IRS1-Akt-GLUT4 signaling pathway.

In T2DM patients, insulin sensitivity decreases in most organs and tissues, accompanied with symptoms like hyperglycemia, hyperlipidemia, hyperinsulinism, and so on [19]. GK rats, a model of T2DM prone rats, were characterized by hyperglycemia, hyperinsulinemia, and IR [20]. Due to these symptoms being similar to T2DM in human, GK rats are widely used in T2DM research. In this study, GK rats fed with high fat and glucose for 4 weeks exhibited a series of T2DM symptoms, including increases of food and water intake as well as urine volume but reduction of body weight. Compared with model group, WSZYF could alleviate the above-mentioned symptoms and better effect was obtained in a higher dose. Moreover, WSZYF also observably decreased fasting blood glucose, glycosylated serum protein, glycosylated hemoglobin, insulin, and IR index compared with those in model group. Meanwhile,* in vitro* results showed that WSZYF could also enhance glucose consumption in IR model of skeletal muscle cells. All of these suggested that WSZYF could regulate glucose metabolism disorders and improve IR in T2DM rats.

There are three signaling pathways mentioned in insulin signal transduction pathways including PI3K pathway, mitogen activated protein kinase pathway, and C-Cbl related protein pathway. Among them, PI3K signaling pathway is the classical one [21–24]. The normal transduction of PI3K signaling pathway is directly related to the normal metabolisms of glucose, fat, and protein. Meanwhile, any abnormalities of PI3K signaling pathway transduction may lead to IR [25]. In the normal process, IRS is phosphorylated when insulin combines with its receptor in the surface of skeletal muscle cell, followed by PI3K activation. The activated PI3K can catalyze phosphatidylinositol 4,5-bisphosphate (PIP2) to PIP3, which activates sequence downstream signaling factor Akt. Phosphorylated Akt translocates GLUT4 to the plasma membrane to uptake glucose into the muscle, which contributes to decreasing plasma glucose concentrations [26]. Therefore, IRS-PI3K/Akt-GLUT4 signaling pathway exerts vital regulative effect in insulin signaling transduction of skeletal muscle. Any disorder in this pathway can reduce the sensitivity of skeletal muscles to insulin, resulting in impaired glucose uptake, utilization, glucose tolerance, and elevated blood glucose level [27]. Compared with control group, the expressions of p-IRS1, p-Akt, and GLUT4 decreased significantly in model group, indicating that insulin signaling transduction of skeletal muscle was abnormal and IR occurred. With the treatment of WSZYF, expressions of the signal molecules mentioned above had been elevated, verifying that WSZYF improved IR via restoring insulin signaling transduction of skeletal muscle.

Protein tyrosine phosphatase-1B is an important target for T2DM treatment and has been proved to play a vital role in the negative regulation of insulin signaling transduction. It promotes the dephosphorylation of insulin receptor and IRS and downregulates insulin signaling transduction, ultimately leading to IR [13]. The expression of PTP1B increased in model group, thus inhibiting IRS1-Akt-GLUT4 signaling pathway and resulting in IR. This was similar to other studies [12, 14]. WSZYF reduced PTP1B expression in a dose-dependent manner, followed by the restoration of IRS1-Akt-GLUT4 signaling pathway and mitigation of IR.

## 5. Conclusion

In summary, we investigated the antidiabetic effect of WSZYF in T2DM animal model and IR model of skeletal muscle in this study. The results demonstrated that WSZYF could regulate glucose mechanism and IR through modulating PTP1B-IRS1-Akt-GLUT4 signaling pathway. This study provides basis for further research on effective components in the treatment of T2DM.
